# Progression of Atrioventricular Conduction Disorder Induced by Radiofrequency Ablation for Hepatocellular Carcinoma

**DOI:** 10.7759/cureus.111444

**Published:** 2026-06-24

**Authors:** Rakuhei Nakama, Miyuki Sone, Masaaki Shoji, Nozomu Ogura, Takuji Okusaka

**Affiliations:** 1 Department of Diagnostic Radiology, National Cancer Center Hospital, Tokyo, JPN; 2 Department of Cardiovascular Medicine, National Cancer Center Hospital, Tokyo, JPN; 3 Department of Hepatobiliary and Pancreatic Oncology, National Cancer Center Hospital, Tokyo, JPN

**Keywords:** atrio-ventricular block, cardiac conduction disorder, clinical case, hepatocellular carcinoma (hcc), radio-frequency ablation

## Abstract

Radiofrequency ablation (RFA) is generally considered a safe treatment for hepatocellular carcinoma. Although cardiovascular complications have been occasionally reported, they are primarily attributed to sedatives and analgesics or the vasovagal reflex. However, a unique case is presented where RFA itself was suspected of inducing a progressive atrioventricular disorder. An 80-year-old male underwent RFA for hepatocellular carcinoma. His electrocardiogram showed a first-degree atrioventricular block on admission. Prior to ablation, fentanyl and propofol were administered. Severe bradycardia developed immediately after initiation of ablation. RFA and propofol infusion were terminated, leading to prompt heart rate recovery. After approximately five minutes, ablation was resumed without propofol, but severe bradycardia recurred. The electrocardiogram during RFA revealed a complete atrioventricular block. Therefore, the procedure was terminated. A 12-lead electrocardiogram performed after the aborted procedure revealed progression to a second-degree AV block (Mobitz type II) requiring pacemaker implantation. The exact mechanism of this effect remains unclear, though progression of the atrioventricular conduction disorder was considered to be induced by RFA. Careful intraoperative patient management is crucial during RFA.

## Introduction

Similar to surgery, radiofrequency ablation (RFA) is a widely used curative treatment for hepatocellular carcinoma (HCC) [1]. In addition, as an advantage, RFA can be performed safely without general anesthesia [2]; thus, it represents a relatively safe treatment procedure for HCC. However, certain potential complications could occur, including hemorrhage, liver abscess, portal vein thrombosis, pleural effusion, and gastric perforation caused by puncture and ablation [3].

Among them, cardiovascular complications must also be focused on. In general, certain sedatives and analgesics are commonly administered during RFA to alleviate procedure-associated pain or anxiety, a process known as "procedural sedation" [4]. Multiple sedatives and analgesics possess vasodilatory properties and could depress circulatory function, often causing adverse reactions such as bradycardia and hypotension [5]. Severe bradycardia and arrhythmia could occur in several cases, potentially leading to cardiac arrest.

In addition, even with such procedural sedation, pain control for RFA can be difficult. Severe pain with ablation causes a vasovagal reflex and often induces bradycardia [6]. Thus, it is necessary to consider the cardiovascular impact during the procedure.

However, because various factors are involved, there are cases where the exact cause of circulatory failure during RFA cannot be identified. This report presents a case in which RFA itself appeared to have induced atrioventricular conduction disturbances, resulting in atrioventricular block progression.

## Case presentation

An 80-year-old man was hospitalized for HCC treatment using RFA. His medical history included percutaneous coronary stenting for angina (left anterior descending branch), chronic renal failure, and pancreatic intraductal papillary mucinous neoplasm. He did not experience any syncopal episodes. The initial electrocardiogram (ECG) on admission revealed a first-degree atrioventricular block without any symptoms (Figure 1).

**Figure 1:**
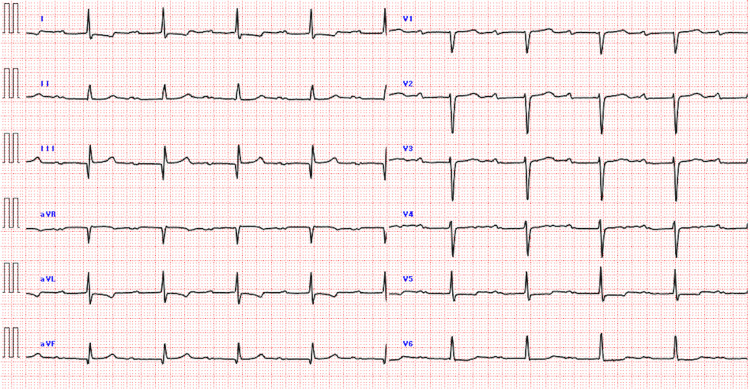
Initial electrocardiography findings on admission This ECG showed a prolonged PQ interval (420 msec). The ECG diagnosis was a first-degree atrioventricular block.

Initially, transcatheter arterial embolization (TAE) was performed to mark the lesion in liver segment 3 (Figure 2), followed by RFA on the following day. The target tumor was punctured percutaneously under ultrasound guidance using a 17-gauge, straight, rigid radiofrequency electrode with a 30-mm exposed tip (Cool-tip RF ablation System E Series; Medtronic, Minneapolis, MN, USA). Proper positioning of the puncture needle was confirmed using computed tomography (Figure 3).

**Figure 2:**
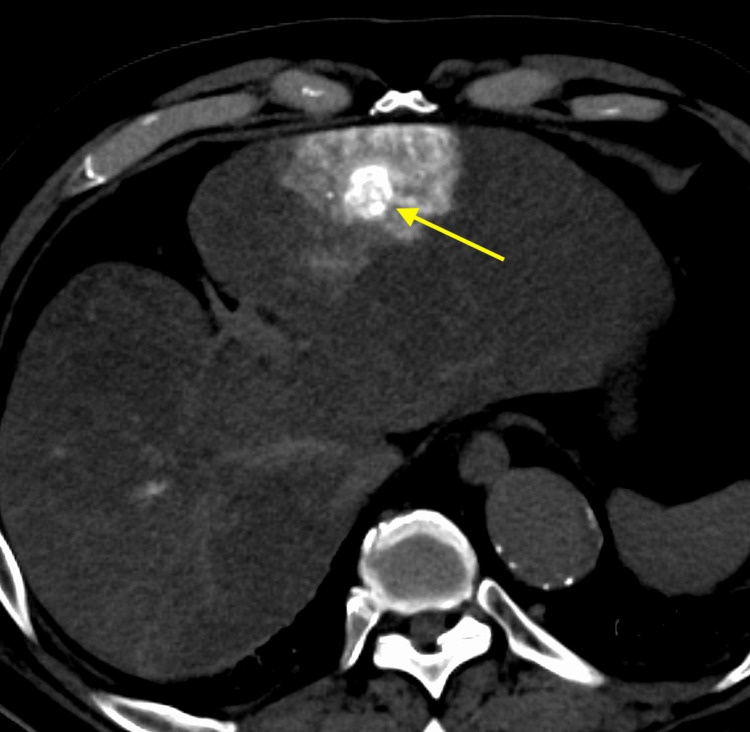
Computed tomography after TAE Appropriate lipiodol deposition was detected on the target lesion in liver segment 3 (arrow). TAE: transcatheter arterial embolization

**Figure 3:**
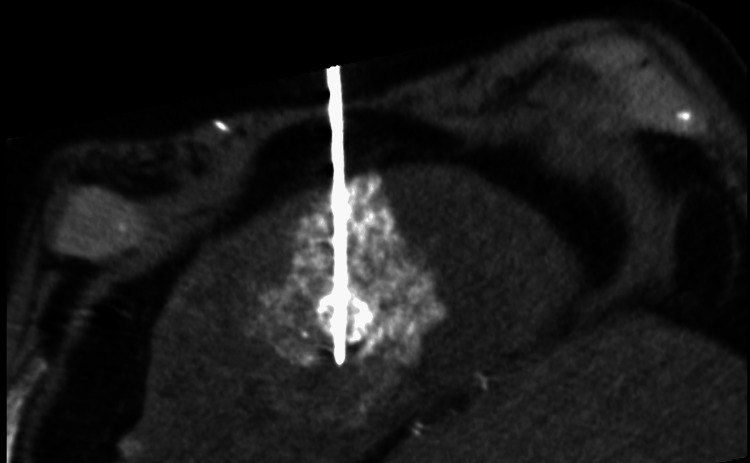
Post-puncture CT image This image demonstrates precise placement of the radiofrequency ablation needle within the center of the tumor.

Prior to the ablation procedure, fentanyl and propofol were administered (fentanyl and propofol: 35 mcg and 35 mg bolus injection, respectively, and 100 mg/h continuous intravenous infusion). Following the confirmation of adequate sedation depth, ablation was initiated at 40W. Immediately after initiation of ablation, severe bradycardia (heart rate of approximately 20 beats per minute) developed promptly, attributed to circulatory suppression, potentially induced by propofol. Therefore, ablation and continuous propofol infusion were terminated, leading to the prompt heart rate recovery (approximately 60 beats per minute) of the patient.

After approximately five minutes, ablation was resumed with fentanyl alone, without propofol. However, severe bradycardia recurred. Monitoring the ECG during the procedure revealed a complete atrioventricular block, considered to be induced by RFA. Therefore, the procedure was terminated. As in the case of the first ablation session, bradycardia improved rapidly. However, the 12-lead ECG performed in the recovery room displayed progression from a first-degree atrioventricular block on admission to a second-degree atrioventricular block (Mobitz block type II) (Figure 4). The patient was transferred to another hospital for a more thorough examination of the cardiovascular system. He underwent an electrophysiological examination by a cardiologist, and finally, pacemaker implantation was performed.

**Figure 4:**
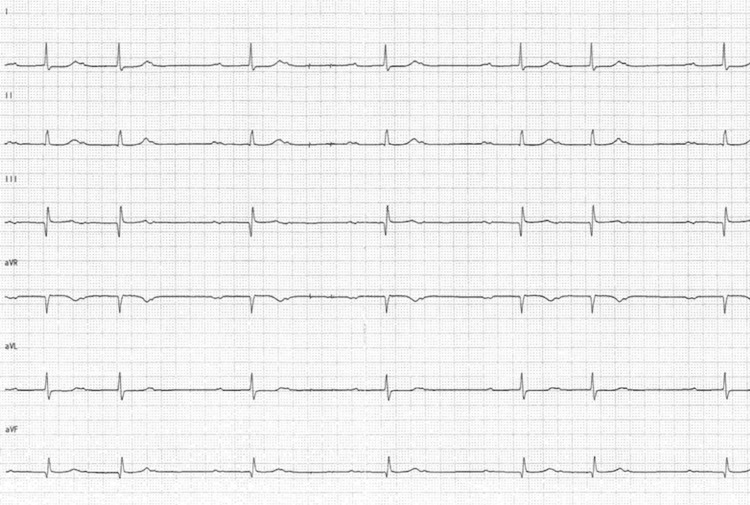
The 12-lead electrocardiography in the recovery room after RFA This ECG showed a QRS deficit without a prolonged PQ interval. ECG diagnosis was a second-degree atrioventricular block / Mobitz block type II. RFA: radiofrequency ablation

## Discussion

This is a case in which RFA potentially triggered the progression of atrioventricular conduction disturbances. To the best of our knowledge, no previous studies have reported that RFA for HCC could cause abnormalities in the conduction system.

As a cardiovascular complication during RFA for HCC, a case of stress (Takotsubo) cardiomyopathy has been reported [7]. However, the mechanism of the occurrence was attributed to the invasive procedure-associated catecholamine release, and not a direct RFA effect. With respect to how RFA affected the conduction system, a report has emerged of unexpected changes in pacemaker function occurring during ablation [8]. However, this represents merely a reaction to the pacemaker and cannot be considered the same as a reaction to the conduction system in humans.

Transcatheter RFA for arrhythmia reportedly caused an atrioventricular block, in which ablation-induced edema around the atrioventricular node is considered a possible cause [9]. Our case involved liver ablation distant from the heart, suggesting an indirect mechanism such as an effect on electrical conduction.

In general, the sedative effects of propofol or fentanyl during the procedure are more likely to cause bradycardia. However, a complete atrioventricular block occurred simultaneously with the start of ablation in both sessions, suggesting reproducibility. In particular, in the second ablation session, sufficient time had elapsed since propofol administration was discontinued. Considering its half-life (i.e., a few minutes), the pharmacological effects of propofol were considered to have almost disappeared. Therefore, RFA might have directly caused the progression of the atrioventricular block.

Furthermore, it has been reported that reflex bradycardia can be induced by mechanisms other than the vasovagal reflex, even when pain is adequately managed [10]. Although reflex bradycardia is generally reversible, the intraoperative bradycardia event may have triggered a permanent conduction abnormality.

One notable feature of this case was that the ECG on admission revealed a first-degree atrioventricular block with prominent PQ interval prolongation. In addition to this patient-specific background, a complex interplay of the direct effects of RFA on the conduction system, pharmacological influences, and various reflex responses may have led to the development of permanent conduction disturbance. However, the precise underlying mechanism through which RFA induces atrioventricular conduction disturbances remains unclear. Further accumulation of cases is required to validate our findings.

## Conclusions

In conclusion, this case highlights that RFA can potentially trigger the development of a progressive and permanent atrioventricular block. While RFA is generally considered a safe procedure, clinicians should remain careful regarding rare but severe complications such as the unexplained adverse events observed in this case. Further studies are required to elucidate the underlying mechanisms.
